# Virtual screening and molecular dynamic simulations of the antimalarial derivatives of 2-anilino 4-amino substituted quinazolines docked against a *Pf*-DHODH protein target

**DOI:** 10.1186/s43042-022-00329-2

**Published:** 2022-08-10

**Authors:** Zakari Ya’u Ibrahim, Adamu Uzairu, Gideon Adamu Shallangwa, Stephen Eyije Abechi, Sulaiman Isyaku

**Affiliations:** Department of Chemistry, Faculty of Physical Sciences, Ahmadu Bello University, P.M.B 1045, Zaria, Nigeria

**Keywords:** Virtual screening, Antimalarial, Docking, Dynamic simulation, 2-Anilino 4-amino substituted quinazolines, And *Pf*-DHODH

## Abstract

**Background:**

The processes of drug development and validation are too expensive to be subjected to experimental trial and errors. Hence, the use of the insilico approach becomes imperative. To this effect, the drug-likeness and pharmacokinetic properties of the ten (10) previously designed derivatives of 2-anilino 4-amino substituted quinazolines were carried out. Their predicted ligand binding interactions were also carried out by docking them against the *Plasmodium falciparum* dihydroorotate dehydrogenase (*Pf-*DHODH) protein target, and the stability of the complex was determined through dynamic simulations. The drug-likeness and pharmacokinetic characteristics were estimated using the online SwissADME software, while the Molegro Virtual Docker (MVD) software was used for molecular docking. And the dynamic simulation was performed for the duration of 100 ns to verify the stability of the docked complex, with the aid of a Schrödinger program, Desmond.

**Results:**

The designed derivatives were all found to pass the Lipinski test of drug likeness, while the pharmacokinetic studies result that the skin permeability and molar refractivity values of the derivatives are both within the limits. In addition, except for derivative C-01, most of the derivatives have strong gastrointestinal absorptions and lack Pgp substrate. Furthermore, no derivative inhibited CYP1A2, CYP2C9, or CYP2C19. The docking studies show the better binding affinities between the ligands and *Pf-*DHODH than those between the atovaquone or chloroquine standards. The derivative C-02, {5-((6,7-dimethoxy-4-((3-nitrobenzyl)amino)quinazolin-2-yl)amino)-2-fluorobenzaldehyde} was found to be the most stable derivative, with a re-rank docking score of − 173.528 kcal/mol and interaction energy of − 225.112 kcal/mol. The dynamic simulation analysis shows that the derivative C-02 forms a stable complex with the protein target over the simulation time.

**Conclusions:**

The ability of these ligands to form hydrogen bonds, as well as various other interactions, was cited as a factor responsible for their better binding affinity. These findings could aid further the development of enhanced antimalarial drugs.

## Research highlights

The research aimed at screening of the antimalarial derivatives of 2-anilino 4-amino substituted quinazolines docked against a *Pf*-DHODH protein target. Hence, some of the highlights are listed below:

1. The developed derivatives were discovered to follow Lipinski's rule of five in all of their properties, making them appropriate for drug treatment.
2. ADME pharmacokinetic properties of the designed derivatives were studied to know the oral bioavailability of the derivatives.
3. The ADME predictions shows the skin permeability of the derivatives to be within the tolerances level, as was their molar refractivity value.
4. The derivatives show high gastrointestinal absorptions in all the derivatives except in derivatives C-01, C-06, C-09, and C-10.
5. The designed derivatives may use in the treatment of other form of malarial with the exception of cerebral malaria since they lack BBB penetration.
6. Furthermore, the docking studies conducted shows *Pf-*DHODH to binds excellently with the designed ligands.
7. Derivative C-02, {5-((6,7-dimethoxy-4-((3-nitrobenzyl)amino)quinazolin-2-yl)amino)-2-fluorobenzaldehyde} (highest re-rank score and interaction energy of − 173.528 kcal/mol and an interaction energy of − 225.112 kcal/mol, respectively), was identified as the most stable of the designed derivatives.
8. The derivative C-04, N4-(3-chloro-5-fluorobenzyl)-N2-(4-fluorophenyl)-6,7-dimethoxyquinazoline-2,4-diamine, was found to be the least stable with − 120.489 and − 191.98 kcal/mol re-rank score and interaction energy, respectively.
9. Derivative C-02 binds with various amino acid residues such as Gly277, Phe278, Asn347, Lys429, Gly506, twice with Ile508, and Ala225 to produce eight (8) conventional hydrogen bonds with bonds lengths 2.7238 Å, 2.4607 Å, 2.3411 Å, 2.3244 Å, 2.0826 Å, 2.9660 Å, 2.0304 Å, and 2.6270 Å, respectively, in addition to two (2) carbon hydrogen bonds with Gly478 and Gly507 with bond distances of 2.4691 and 2.7052 Å, respectively.
10. The dynamic simulations results shows derivative C-02 forms a stable complex with the protein target.

## Introduction

Malaria is one of the top ten global infectious diseases that require prompt treatment, especially in sub-Saharan Africa and over 90 nations [21, 22]. In 2021, the World Health Organization (WHO) reported the occurrence of over 241 million malaria cases worldwide, with an estimated death rate of 627,000 deaths in 2020 [7]. Among the four Plasmodium species (*Plasmodium falciparum*, *P. ovale*, *P. vivax*, and *P. malariae*), *Plasmodium falciparum* (*P. falciparum*) handles over 90% of malarial disorders [12].

Despite massive attempts, vaccine development has fallen short, leaving chemotherapy as the major means of preventing the disease's spread. Artemisinin and chloroquine derivatives are the two main types of medications used to treat malaria because of their efficacy, safety, and accessibility [33]. The resistance of Plasmodium parasites to this treatment is a major roadblock, prompting researchers to look for other molecular targets and better possible treatments [11].

In contrast to human cells, which salvage preformed pyrimidine based as well as pyrimidine production from the host cell through the de novo pathway, *P. falciparum* parasites rely on nucleotide synthesis through the de novo process to provide the essential precursor for DNA and RNA formation. The metabolic pathways of *Plasmodium* are not the same as those of human hosts. Hence, the de novo or salvage pathway becomes the only route to biosynthesize the purine, pyrimidines, and even the nucleotides [19]. Dihydroorotate dehydrogenase catalyzes the oxidation of l-dihydroorotate (DHO) to create orotate, which is the fourth and rate-limiting step in the pyrimidine biosynthesis pathway [32].

Heterocyclic rings with nitrogen and sulfur are therapeutically and pharmacologically reported active and are of particular interest [18]. Quinazoline derivatives are heterocyclic molecules with nitrogen fixed in the ring that exhibit antiviral, antidiabetic, antimalarial, antioxidant, and anti-inflammation characteristics, to mention a few [3, 6, 10, 14, 20]. In recent years, several medications, such as 2-anilino 4-amino substituted quinazolines, have shown antimalarial efficacy [9]. While no unique pattern has been established as being responsible for their antimalarial action, their reported potency may be because of replacement at specific places. Also, the interactions of quinazolin-2,4-dione with (Pf-DHODH) were described by Haredi Abdelmonsef and colleagues [12]. The core scaffold of *Plasmodium falciparum* dihydroorotate dehydrogenase was quinazolin-2,4-dione, which was coupled to a nitrogen-containing heterocyclic structure via acetyl/amide linkages.

A never-ending series of studies were reported on the molecular docking and pharmacokinetic properties of antimalarial medications. The works of Qidwai [24], Alzain et al. [2], Tahghighi [30] and Shah [26] are only a few examples. The current study examines the drug-likeness and pharmacokinetic properties of the generated derivatives, as well as docking them against the *Plasmodium falciparum* dihydroorotate dehydrogenase, *Pf-*DHODH protein, to predict ligand binding interactions and explain why they occur.

## Material and methods

### Drug likeness and ADME prediction

This investigation used derivatives of 2-anilino 4-amino substituted quinazolines (Table 1) from our earlier work [17]. As drug-like qualities, the sizes of the molecules, the amounts of hydrogen bond donors and acceptors (Lipinski parameters), as well as additional properties like the topological polar surface area (TPSA) and the number of rotatable bonds [29], were all determined. Violating two or more Lipinski factors cast doubt on the intended derivatives' bioavailability as prospective pharmaceuticals [23]. The most significant pharmacokinetic aspects of any medicine are its ADME (absorption, distribution, metabolism, and excretion) [16]. These properties are measured using molar refractivity (MR), the number of rotatable bonds (*n*Rotb), log of skin permeability (log Kp), blood–brain barrier (BBB) penetration, permeability glycoprotein (Pgp) substrate, gastrointestinal (GI), as well as cytochrome P450 inhibitor enzymes (CYP1A2, CYP2C9, and CYP2C19) of 2-anilino 4-amino, substituted quinazolines derivatives. The online SwissADME software (http://www.swissadme.ch) is used to predict these pharmacokinetic properties.

**Table 1:** Molecular formula, IUPAC Names, and structures of the designed derivatives of 2-anilino 4-amino substituted quinazolines

S/N	Formula	IUPAC name	Structure
C-01	C_24_H_20_F_4_N_4_O_2_	N2-(4-fluorophenyl)-6,7-dimethoxy-N4-(3-(trifluoromethyl)benzyl)quinazoline-2,4-diamine	
C-02	C_24_H_20_FN_5_O_5_	5-((6,7-dimethoxy-4-((3-nitrobenzyl)amino)quinazolin-2-yl)amino)-2-fluorobenzaldehyde	
C-03	C_23_H_19_BrF_2_N_4_O_2_	N4-(3-bromo-5-fluorobenzyl)-N2-(4-fluorophenyl)-6,7-dimethoxyquinazoline-2,4-diamine	
C-04	C_23_H_19_ClF_2_N_4_O_2_	N4-(3-chloro-5-fluorobenzyl)-N2-(4-fluorophenyl)-6,7-dimethoxyquinazoline-2,4-diamine	
C-05	C_23_H_19_BrFN_5_O_4_	N4-(3-bromo-5-nitrobenzyl)-N2-(4-fluorophenyl)-6,7-dimethoxyquinazoline-2,4-diamine	
C-06	C_23_H_19_F_2_N_5_O_4_	N4-(3-fluoro-5-nitrobenzyl)-N2-(4-fluorophenyl)-6,7-dimethoxyquinazoline-2,4-diamine	
C-07	C_24_H_20_FN_5_O_2_	3-(((2-((4-fluorophenyl)amino)-6,7-dimethoxyquinazolin-4-yl)amino)methyl)benzonitrile	
C-08	C_23_H_20_F_2_N_4_O_2_	N4-(3-fluorobenzyl)-N2-(4-fluorophenyl)-6,7-dimethoxyquinazoline-2,4-diamine	
C-09	C_23_H_20_BrFN_4_O_2_	N4-(3-bromobenzyl)-N2-(4-fluorophenyl)-6,7-dimethoxyquinazoline-2,4-diamine	
C-10	C_23_H_20_FIN_4_O_2_	N2-(4-fluorophenyl)-N4-(3-iodobenzyl)-6,7-dimethoxyquinazoline-2,4-diamine	

### Structure validation protein

**Fig. 1:**
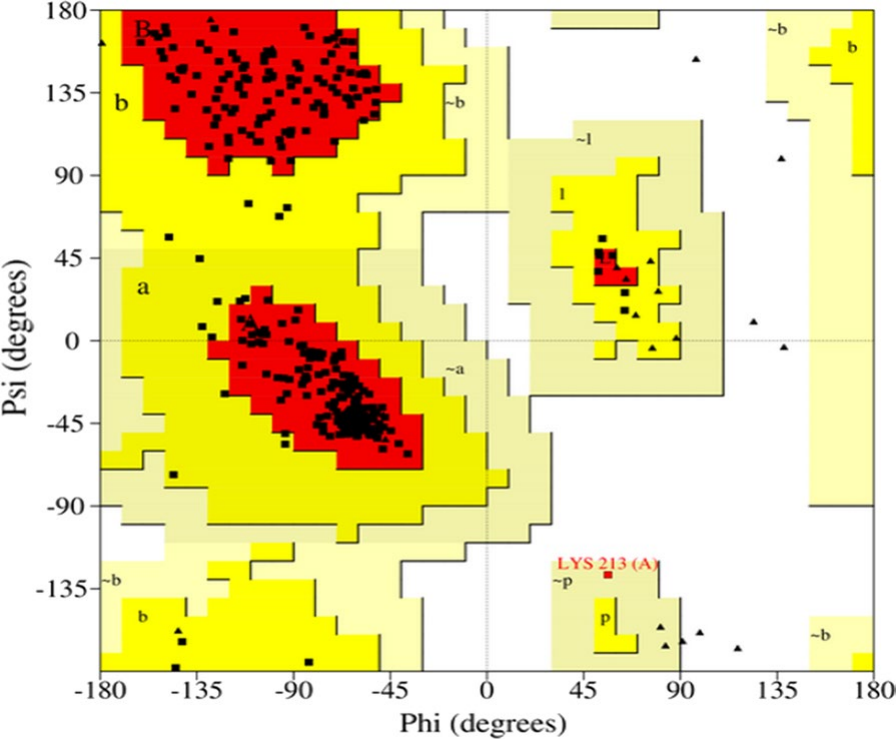
Ramachandran plot analysis of *Plasmodium falciparum* dihydroorotate dehydrogenase, where the red region indicates the favored region, the yellow region for allowed and light yellow shows the generously allowed region, and white for the disallowed region. Phi and Psi angles determine torsion angles

To validate the protein structure, the pdb file format of the *Pf-*DHODH was uploaded into the procheck tool (https://saves.mbi.ucla.edu/) [13] to generate both the Ramachandran plot (Fig. 1) and its statistics (Table 2). The Ramachandran plot was used to examine the quality of a protein or an experimental structure, while the Ramachandran plot statistics provide information on the total number of amino acid residues found in the favorable, allowed, and disallowed regions [15].

**Table 2:** Ramachandran plot of *Plasmodium falciparum* dihydroorotate dehydrogenase from *P. falciparum* strain

Ramachandran plot statistics	*Plasmodium falciparum* dihydroorotate dehydrogenase	
	Residue	%
Residues in most favored regions [A, B, L]	314	93.50
Residues in additional allowed regions [a, b, l, p]	21	6.20
Residues in generously allowed regions [~ a, ~ b, ~ l, ~ p]	1	0.30
Residues in disallowed regions	0	0.00
Number of non-glycine and non-proline residues	336	100.00
Number of end-residues (excl. Gly and Pro)	4	
Number of glycine residues (shown as triangles)	27	
Number of proline residues	11	
Total number of residues	378	

### Ligand preparation

The derivatives were sketched in ChemDraw Ultra 12 and then imported to spartan'14 version 1.1.2 software, where they were fully optimized using a density functional theory (DFT/B3LYP/6-31G*) to achieve the molecules' ideal structures. The ligands were configured and saved as PDB files, which were then imported into the Molegro Virtual Docker (MVD) and prepared with the preparation wizard.

### Protein preparation

**Fig. 2:**
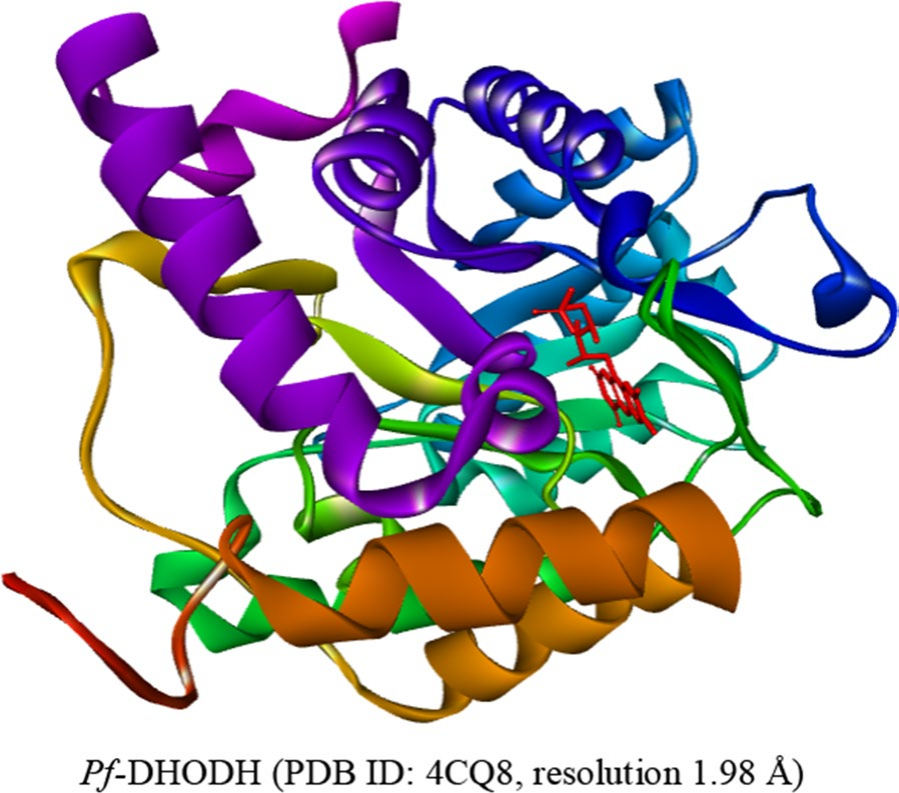
Derivatives of 2-anilino 4-amino substituted quinazolines (red) in the binding sites of *Pf-*DHODH

The RCSB protein data library was used to derive the three-dimensional structures of the *Plasmodium falciparum* dihydroorotate dehydrogenase, *Pf-*DHODH, in Fig. 2 (PDB ID: 4CQ8, resolution 1.98 Å). The MVD's protein preparation wizard was used to restore charges and any hydrogen that had been lost during the procedure. The MVD program's built-in cavity algorithm was used to look for the protein's binding pockets.

### Docking specifications

The Plant score Grid with over 0.3 Å grid resolution was selected as the scoring function for the study. The docking radius was then set to 18, which covered over 90% of the protein cavities found. Besides ticking the *energy minimization*, *constraining poses to the cavity*, and *optimizing H-bonds* boxes, the MolDock SE searching algorithm was also chosen. The iterations, the population size, and the energy threshold were all respectively set to a maximum of 1500, 50, and 100.00, leaving the *Tries values for the min*, *fast*, and *max tries* to be 10, 10, and 30, respectively. The default values for the Max phase and neighbor distance factor are, respectively, 300 and 1.00. In the pose clustering dialog box, the *energy threshold* was also activated.

### Docking of molecules

Molecular docking experiments are used to determine the orientation and molecular interactions between derivatives of 2-anilino 4-amino substituted quinazolines and their protein targets. For molecular interactions and research, the Molegro Virtual Docker (MVD) tool was utilized to import the generated derivatives into the binding sites of the particular protein targets.

### Docking validation

The molecular docking process was confirmed by removing the co-crystallized ligand from the protein and re-docking it into the protein's active site. The root-mean-square deviation (RMSD) between the superimposed ligands was computed using the docked conformation ligand perfectly superimposed on the co-crystallized ligand. The RMSD should be less than 2 Å for the docking process to be verified [28].

### Molecular dynamic simulations

A package of the Schrödinger program named Desmond was used to carry out the molecular dynamic simulations [5]. This begins by placing the selected ligand-*Pf*-DHODH protein complex in an SPC (single point charge) water box extending 10 Å beyond any of the complex's atoms. To neutralize charges, counter ions (30 Na^+^ and 35 Cl^−^) were introduced. To simulate physiological conditions, the salt content was fixed to 0.15 M sodium and chloride ions. The simulation was carried out in the NPT ensemble at a temperature of 300 K and a pressure of 1.63 bar for 100 ns, with recording intervals of 1.2 ps and 10 ps for the energy and trajectory, respectively. The OPLS 2005 force field was used to execute the simulations. Desmond simulation interaction diagram tool in Maestro was used to create plots and figures.

## Results

### Drug likeness and ADME prediction

The drug-likeness of the ten designed derivatives of 2-anilino 4-amino, substituted quinazolines were tested using the Lipinski parameters in addition to other parameters as given in Table 3, while their pharmacokinetic properties were determined in form of ADME screening and are presented in Table 4.

**Table 3:** Lipinski and other parameters of 2-anilino 4-amino substituted quinazolines designed compounds

S/N	Lipinski’s parameters					Other parameters	
	MW (≤ 500 Da)	Ilogp (< 5)	#H-bond acceptors (≤ 10)	#H-bond donors (≤ 5)	#Lipinski violations	TPSA (< 140 Å^2^)	nRotB (≤ 10)
C-1	472.43	4.22	8	2	0	68.30	8
C-2	429.45	3.90	6	2	0	92.09	7
C-3	422.43	3.65	6	2	0	68.30	7
C-4	483.33	4.38	5	2	0	68.30	7
C-5	530.33	4.31	5	2	1	68.30	7
C-6	477.44	3.24	8	2	0	131.19	9
C-7	501.32	4.36	6	2	1	68.30	7
C-8	456.87	4.21	6	2	0	68.30	7
C-9	528.33	3.90	7	2	1	114.12	8
C-10	467.42	3.65	8	2	0	114.12	8

*MW* molecular weight, *LogP* log of octanol/water partition coefficient, *HBA* hydrogen bond acceptor counts, *HBD* hydrogen bond donor counts, *TPSA* topological polar surface area, *nRotB* the number of rotatable bonds

**Table 4:** Pharmacokinetics properties of the designed derivatives of 2-anilino 4-amino substituted quinazolines

S/N	MR	log Kp (cm/s)	GI absorption	BBB permeant	Pgp substrate	CYP1A2 inhibitor	CYP2C19 inhibitor	CYP2C9 inhibitor
C-1	120.82	− 4.82	Low	No	Yes	Yes	Yes	Yes
C-2	120.53	− 5.38	High	No	No	Yes	Yes	Yes
C-3	115.77	− 5.06	High	No	No	Yes	Yes	Yes
C-4	123.52	− 5.02	High	No	No	Yes	Yes	Yes
C-5	128.53	− 5.33	High	No	No	Yes	Yes	Yes
C-6	130.03	− 5.97	Low	No	No	Yes	Yes	Yes
C-7	123.47	− 5.06	High	No	No	Yes	Yes	Yes
C-8	120.78	− 4.83	High	No	No	Yes	Yes	Yes
C-9	132.34	− 5.41	Low	No	No	Yes	Yes	Yes
C-10	124.60	− 5.46	Low	No	No	Yes	Yes	Yes

*MR* molar refractivity, *log Kp* log of skin permeability, *GI* gastrointestinal absorption, *BBB* blood–brain barrier, *Pgp* penetration permeability glycoprotein substrate, *CYP450* cytochrome P450 enzymes; CYP1A2, CYP2C9, and CYP2C19 inhibitors

### Molecular docking

Molecular docking was conducted to determine the binding affinities between the designed derivatives and their protein target and the number of hydrogen bonds within a docking pose as reflected in Table 5. The details of the hydrogen bonding between the protein receptor and five of the most active ligands are shown in Table 6, while Table 7 displays the 2–3D docking poses of the interactions

**Table 5:** MolDock score, re-rank score, no. of H-bond(s), interactions and H-bond energies between the *Pf-*DHODH and the designed derivatives of 2-anilino 4-amino substituted quinazolines

S/N	MolDock score (kcal/mol)	Re-rank score (kcal/mol)	No. of H-bond (s)	Interaction energy (kcal/mol)	Hydrogen bond energy (kcal/mol)
C-01	− 179.7780	− 147.6690	3	− 203.3190	− 3.7483
C-02	− 208.4770	− 173.5280	8	− 225.1120	− 12.8010
C-03	− 174.8540	− 136.2250	4	− 193.0090	− 5.1106
C-04	− 177.9810	− 120.4890	3	− 191.9800	− 7.8548
C-05	− 164.4220	− 136.3650	3	− 193.2480	− 6.8817
C-06	− 185.3780	− 148.4460	4	− 202.2830	− 4.1418
C-07	− 174.6810	− 139.8030	3	− 191.4220	− 5.8534
C-08	− 179.3480	− 144.1550	1	− 192.1280	− 1.7166
C-09	− 168.5140	− 134.5130	4	− 187.1590	− 7.0942
C-10	− 158.1200	− 129.0690	2	− 179.6200	− 4.5687
Atovaquone	-139.7880	− 118.8660	1	− 163.4000	− 2.4935
Chloroquine	− 129.7420	− 106.7580	0	− 146.2770	− 1.6746

**Table 6:** Hydrogen bonding details between the protein receptor and five of the most active ligands

S/N	No. of H-bond(s)	H-binding ligand		Residue	H-binding receptor		H-bond distance (Å)
		Element	Type		Element	Type	
C-2	8	O	A	Gly277	H	D	2.7238
		O	A	Phe278	H	D	2.4607
		O	A	Asn347	H	D	2.3411
		N	A	Lys429	H	D	2.3244
		O	A	Gly506	H	D	2.0826
		F	A	Ile508	H	D	2.9660
		O	A	Ile508	H	D	2.0304
		H	D	Ala225	O	A	2.6270
C-6	4	O	A	His185	H	D	2.4212
		F	A	Cys276	H	D	2.5968
		N	A	Lys429	H	D	2.6870
		F	A	Ile508	H	D	2.4380
C-1	3	F	A	His185	H	D	2.6481
		N	A	Lys429	H	D	2.5974
		F	A	Ile508	H	D	2.3874
C-8	1	F	A	Ile508	H	D	2.1562
C-7	3	N	A	Lys429	H	D	2.2420
		O	A	Asn458	H	D	2.6898
		F	A	Ile508	H	D	2.5555

**Table 7:**
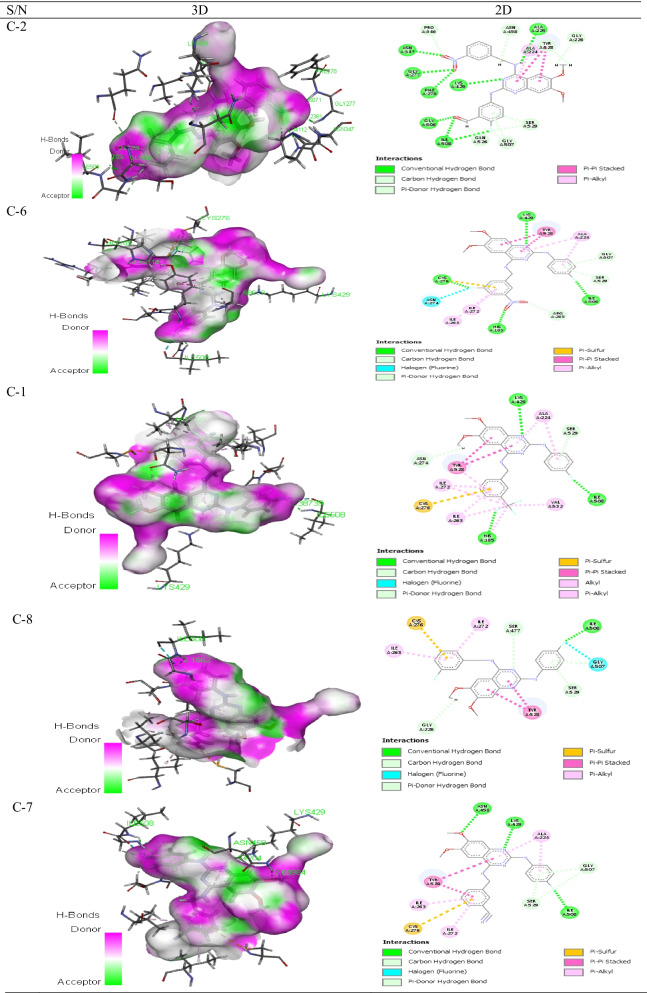
Three-dimensional and two-dimensional docking poses of the five most active compounds

### Dynamic simulation

**Fig. 3:**
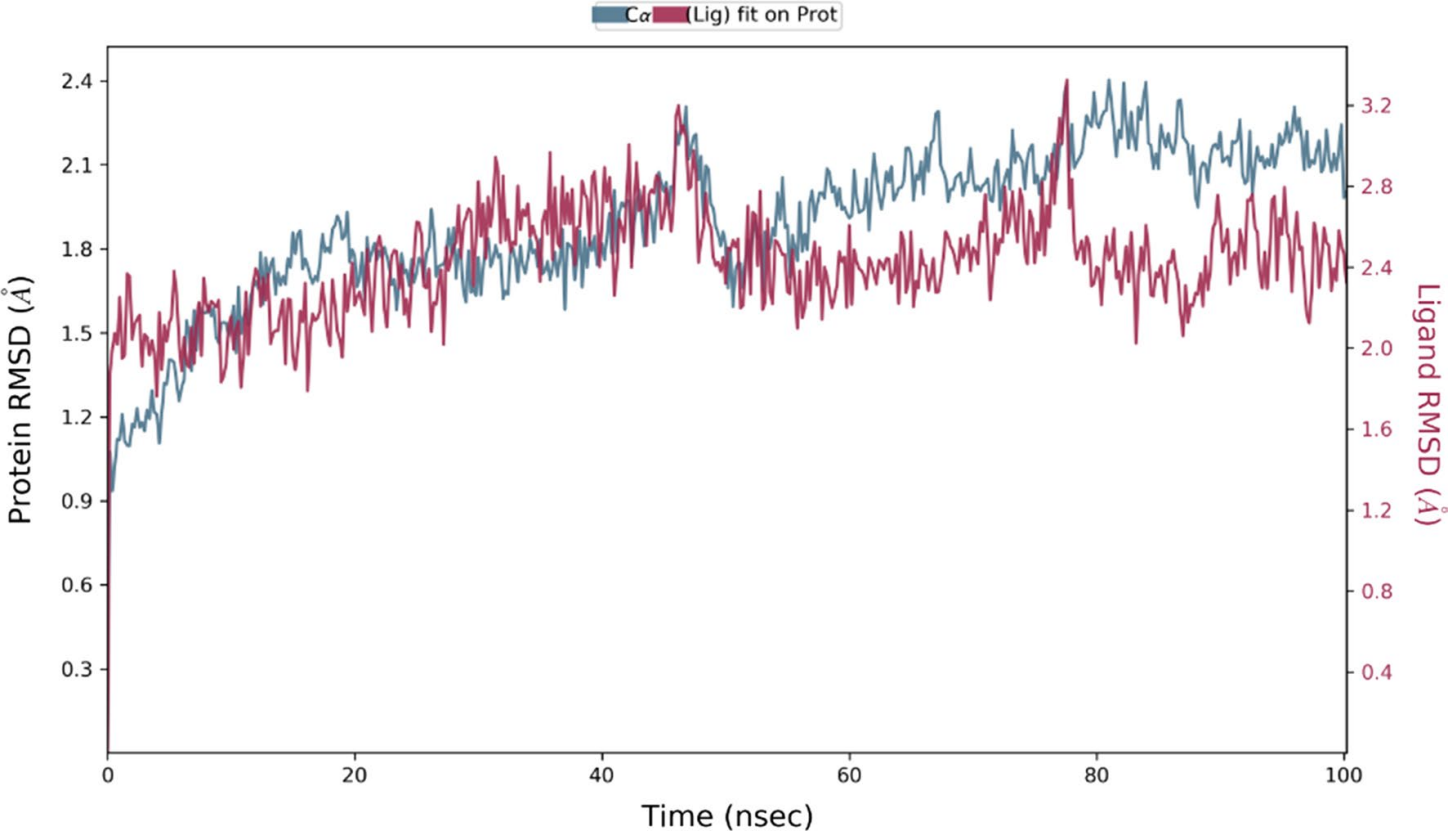
RMSD plot obtained for derivative C-02-*Pf*-DHODH protein (PDB ID: 4CQ8, resolution 1.98 Å) complex

**Fig. 4:**
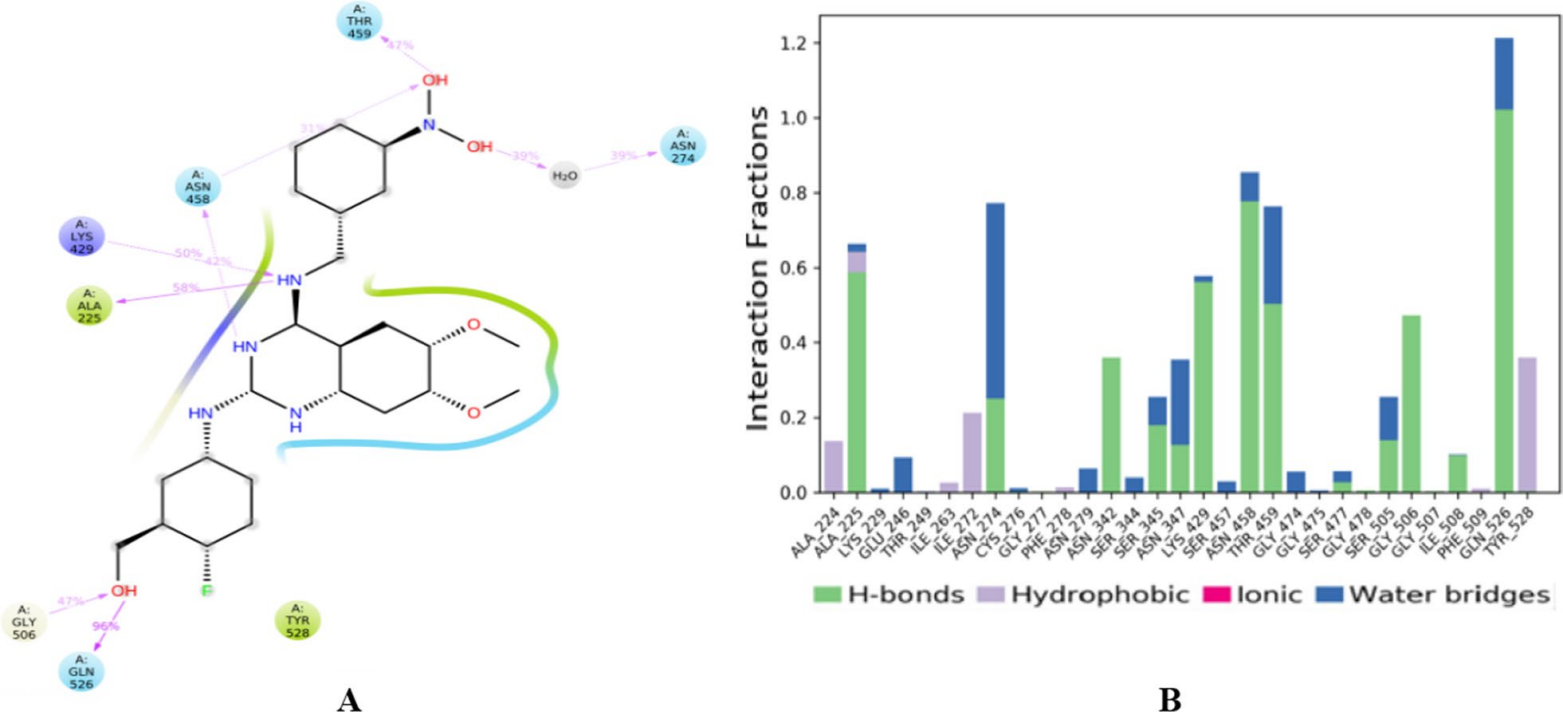
Molecular interaction analysis and type of contact with *Plasmodium falciparum* dihydroorotate dehydrogenase, after MD simulation. The interaction of derivative C-02 atoms with binding site residue of *Pf-*DHODH (4CQ8) (**A**). Normalized stacked bar chart of *Pf-*DHODH binding site residues interacting with derivative C-02 (**B**) through hydrogen bond, hydrophobic and ionic interactions, and water bridges

**Fig. 5:**
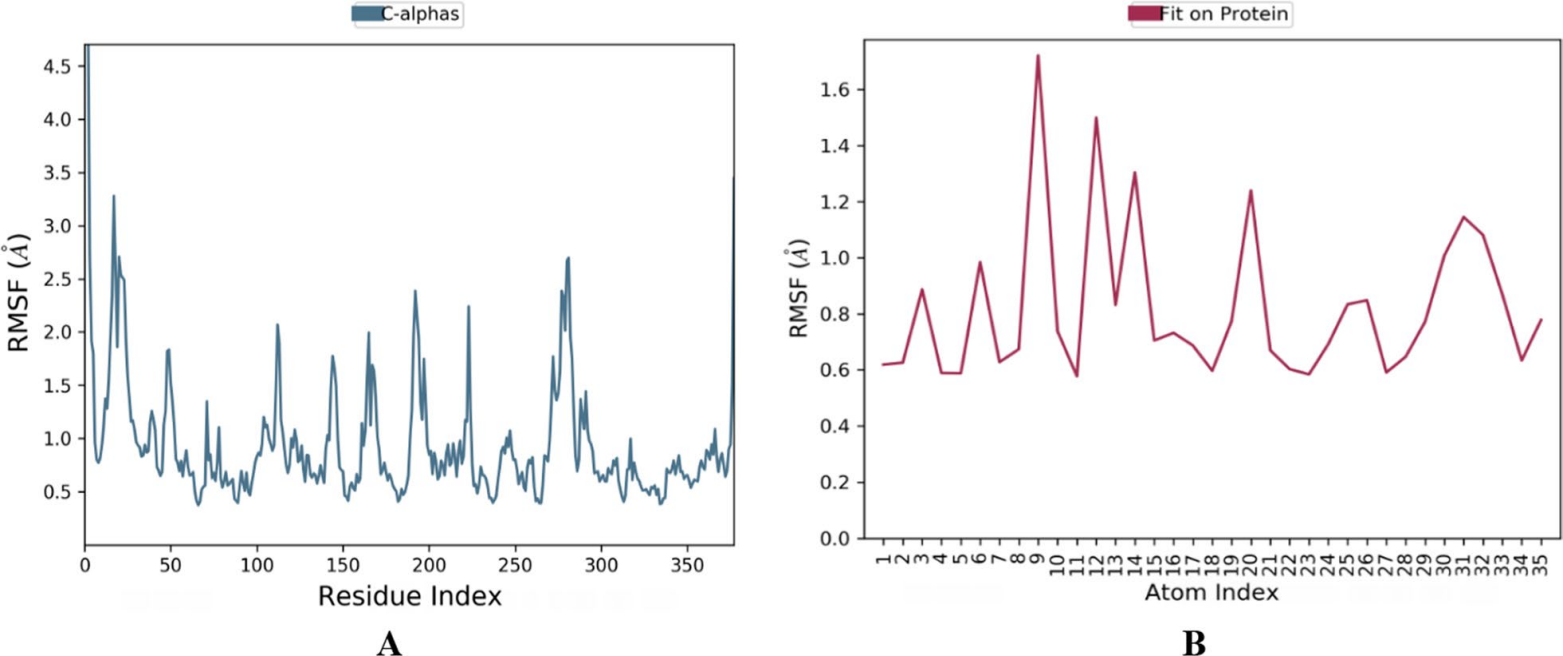
Root-mean-square fluctuations (RMSF) plot for **A** protein **B** ligand

**Fig. 6:**
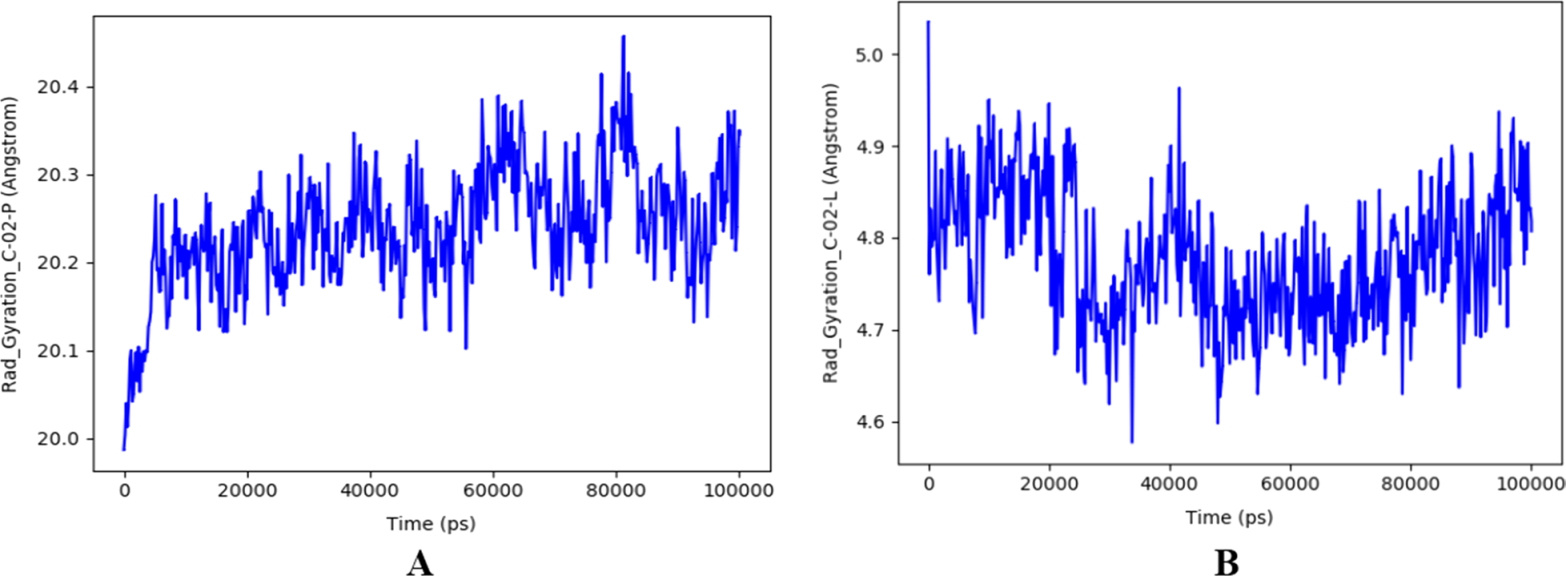
Radius of gyration showing compactness for **A** *Pf-*DHODH **B** ligand, derivative C-02 showing a tolerable deviation during the simulation period

The stability of the selected docked complex was tested through dynamic simulation. With the RMSD plot obtained for derivative C-02-*Pf*-DHODH protein shown in Fig. 3, the molecular interaction analysis and type of contact are shown in Fig. 4. Furthermore, the root-mean-square fluctuations (RMSF) plot for protein ligand and the radius of gyration showing compactness for the protein as well as that of the ligand are shown in Figs. 5 and 6, respectively.

## Discussion

### Drug-likeness and ADME prediction

The results in Table 3 show that besides obeying Lipinski's rule of five (Ro5), all derivatives supported the Veber parameters. Their molecular weight, MW ≤ 500 Da (except C-5, C-7, and C-9), log of octanol/water partition coefficient, ILog*P* < 5, hydrogen bond acceptor (HBA) counts, < 5, hydrogen bond donor (HBD) counts, < 10 (Table 3) showing the excellent drug-like properties of the designed derivatives [25]. Additional parameters, such as topological polar surface area (TPSA) and the number of rotatable bonds (nRotB) were also determined. The TPSA values range between 68.30 and 114.12 Å^2^ values (Table 3) which are greater than 60 Å^2^ but less than 140 Å^2^. Being greater than 60 Å^2^ indicates poor blood–brain barrier penetration, the excellent intestinal absorption is reflected in the less than 140 Å^2^ values [27]. The number of rotatable bonds (nRotB) measures the molecular flexibility of the molecule, with a value within ≤ 10. The designed derivatives have their estimated nRotB ranges between 7 and 9. No derivative was found to violate more than one Lipinski parameter, indicating that the proposed derivatives have good oral absorption. The log of octanol/water partition coefficient (LogP), molecular weight (MW), and topological polar surface area (TPSA) values are all factors of both membrane permeability and oral bioavailability. The in silico ADME values determined (Table 4) revealed the skin permeability (log Kp) of the design derivatives to be within − 4.83 to − 5.97 cm/s, which is within the acceptable range of − 8.0 to − 1.0 cm/s [8]. Molar refractivity is the measure of the volume occupied either by an atom or a group of atoms [1]. The designed derivatives have their molar refractivity values ranging from 115.77 to 132.34, and this falls between the recommended range of 40–130 [4] with C-9 as the only exception. The designed derivatives show high gastrointestinal absorptions except in C-1, C-6, C-9, and C-10, while the BBB investigations (Table 4) show the designed derivatives lacking the ability to penetrate BBB. Hence, the use of derivatives in the treatment of cerebral malaria is rendered ineffective. Substances can enter the cell via active transport or passive diffusion, and they can be effluxed through the permeability glycoprotein (Pgp). Except for C-01, which binds to molecules to efflux, the derivatives were all found to lack Pgp substrate. Table 4 shows the findings of the inhibitory prediction of three Cytochrome P450 (CYP) isoforms; CYP1A2, CYP2C9, and CYP2C19. The drug likeliness and in silico ADME predictions made in this study, as well as those made in investigations with quinazolines-based EGFR inhibitors [31], all passed the test.

### Molecular docking

Molecular docking studies of the derivatives were carried out in the binding pockets of *Pf-*DHODH to determine the protein that will bind better with the ligands. The docking results showing the binding affinity between the ligands with *Pf-*DHODH are shown in Table 5. As shown, the re-rank scores of the derivatives were found to be lower than those of atovaquone and chloroquine standards, a fact supported by the lower binding energies of the ligands with *Pf-*DHODH than with the standards. The high re-rank scores between the ligands and *Pf-*DHODH receptor show an excellent binding mode between them. Derivative C-02, {5-((6,7-dimethoxy-4-((3-nitrobenzyl)amino)quinazolin-2-yl)amino)-2-fluorobenzaldehyde} was found to possess the lowest re-rank score and interaction energies of − 173.5280 and − 225.1120 kcal/mol respectively as reflected in Table 6. The derivative binds with various amino acid residues, where eight of such interactions were with Gly277, Phe278, Asn347, Lys429, Gly506, twice with Ile508, and Ala225 residues to produce eight (8) conventional hydrogen bonds with bonds lengths 2.7238 Å, 2.4607 Å, 2.3411 Å, 2.3244 Å, 2.0826 Å, 2.9660 Å, 2.0304 Å, and 2.6270 Å, respectively, as shown in Table 6. The derivative C-02 also shows seven (7) other interactions leading to carbon-hydrogen bonds and an additional two (2) resulting in Pi-donor hydrogen bonds. These docking results were found to be better than those derived from similar research with benzamide derivatives acting on the same protein target, *Pf-*DHODH [32]. The carbon–hydrogen bonds were with Pro346, Gly507, Ser529, Gly226, Tyr528, Asn458, and Gln526 amino acid residues, while the Pi-donor hydrogen bonds were with Gly507, Ser529 as shown in Table 7. The hydrogen bonds as well as other hydrophobic interactions between the derivative and the receptor could be responsible for the high binding affinity of the derivative. As with derivative C02, the interactions of the next four most active derivatives with the various amino acid residues as well as their 2–3D relation are also shown in Tables 5 and 6. This in silico research revealed the stability of the derived derivatives as a function of hydrogen bonding besides various other interactions with several amino acid residues of the receptor. Hence, derivative C-02 will inhibit *P. falciparum* better than any other derivatives. The docking validation was carried out to determine the docking procedure by measuring the deviation of the re-docking output from the original docking position. The variance is expressed as a root-mean-square deviation (RMSD) value of 0.793 Å. As a result, the docking procedures are validated and can dock the proposed ligands.

### Dynamic simulation

The stability of the proton-ligand complex was assessed in molecular dynamic simulation through the dynamic study of the behavior of the molecular system. As a result, the docked complex of derivative C-02, which had the lowest docking score of − 173.528 kcal/mol, was used for molecular dynamics simulations using the OPLS_2005 force field. The root-mean-square deviation (RMSD), root-mean-square fluctuation (RMSF), and radius of gyration data as a function of time were used to analyze the molecular dynamics simulations. Figure 3 represents the plot of the root-mean-square deviation (RMSD) of the derivative C-02-*Pf*-DHODH protein (PDB ID: 4CQ8) complex. For the 100-ns simulation time, there is no significant conformational change in the protein structure, showing that the complex is stable. The *Pf-*DHODH’s RMSD was plotted on the left *Y*-axis, while that of the ligand aligning on the protein backbone was plotted on the right *Y*-axis. The complex tends to be stabilized during the course of simulation with respect to the reference frame at time 0 ns. Slight fluctuations can however be seen from the 60 ns. The fluctuation is insignificant, ranging from 0.4 to 1.2 Å (i.e., are within the permissible range of 1–3 Å), showing that the protein has not undergone a severe conformational transformation. The residue interactions of *Pf-*DHODH with derivative C-02 are shown in Fig. 4A. It shows that all the interactions of docked pose were retained during the simulation time of 100 ns, i.e., molecular interactions with residues Thr459, Asn274, Asn458, Lys429, Ala225, Gly526, and Tyr528. Figure 4B reflects the derivative C-02-*Pf-*DHODH contacts as stacked bar charts normalized throughout a 100-ns trajectory. The hydrogen bonds, hydrophobic, and ionic interactions, and water bridges are the various categories of ligand–protein contact. During the dynamic simulation, hydrogen bonds, hydrophobic interactions, and water bridges are the main interactions. The hydrogen bonds observed in the docked posed earlier (Lys429 and Ala225) were kept during the dynamic simulation. The root-mean-square fluctuation (RMSF) plot (Fig. 5) measures the fluctuations of every atom in the local domain of the protein and the effect of binding compounds. The protein RMSF (Fig. 5A) shows moderate fluctuations in the binding site residues with an average RMSF value of 1.5 Å, which indicates a lack of significant changes along the protein chain. The ligand RMSF (Fig. 5B) shows a fluctuation ranging between 0.8 and 1.7 Å reflecting the lack of changes in the ligand atom positions. The radius of gyration (Rg) calculates the mass of atoms about the mass of the complex's center. The Rg data implies that ligand movement inside the binding site influences protein structural compactness. The graph of the radius of gyration of protein (Fig. 6A) shows the Rg value of the protein was initially low within 19.9–20.1 Å and then stabilizes within 2.1–2.3 Å for a long period of simulation time. For the ligand (Fig. 6B), the Rg value was initially 4.8–5.0 Å before stabilizing on an average of 4.8 Å. The graphs also show low fluctuation, showing that the *Pf-*DHODH-C-02 complex is in a compact state and does not deviate significantly, hence had better interaction during the ligand simulation.

The techniques employed in this research has found various applications in other antimalarial derivatives such as in the top 10 hits in Alzain's research [2] subjected to ADME calculation. Where, all ten of the compounds show favorable ADME characteristics that will aid in future studies and validations. All of the top 10 compounds fall inside the permitted ranges for absorption, solubility, permeability, and the Lipinski rule and have values that are comparable to those of the reference ligand and DSM265 as well. In the work of Qidwai [24], all of the derivatives of antimalarial compounds have strong binding affinities for the parasite proteins plasmepsin-2 and falcipain-2, according to the docked poses (hemoglobin digesting enzymes). The QSAR model, oral bioavailability, ADME, and toxicity risk assessments indicated that molecules N1, N2, N8, N30, N33, and N39 possess better drug-like qualities than Artemisinin and DHA.

## Conclusion

The eleven (11) designed derivatives of 2-anilino 4-amino substituted quinazolines were screened for their drug-likeness, pharmacokinetic, docking abilities, and as well as dynamic simulation. While the drug-likeness test revealed all the designed derivatives to have passed Lipinski's rule of five (Ro5), the ADME predictions show both the derivatives' skin permeability and molar refractivity values to be within the tolerances limit. Also, the designed derivatives show high gastrointestinal absorptions in all the derivatives except in derivatives C-01, C-06, C-09, and C-10. No derivative was found to inhibit any of CYP1A2, CYP2C9, or CYP2C19 isoforms of Cytochrome P450. Furthermore, the low re-rank scores from the docking investigations are reflections of the several spectacular interactions between the ligands and *Pf-*DHODH. The ligand derivative C-02, {5-((6,7-dimethoxy-4-((3-nitrobenzyl)amino)quinazolin-2-yl)amino)-2-fluorobenzaldehyde} poses the lowest re-rank score of − 173.5280 kcal/mol and showed excellent interactions with several amino acid residues such as Gly277, Phe278, Asn347, Lys429, Gly506, twice with Ile508, and Ala225 besides many other interactions. The dynamic simulation analysis shows that the derivative C-02 forms a stable complex with the protein target over the simulation period of 100 ns. As a result, the in silico designed derivatives show the potential to bind to the receptor more efficiently than the other 2-anilino 4-amino substituted quinazoline derivatives.

## Data Availability

The datasets used for analysis during these studies were included in this published study.
